# Overhead squat assessment reflects treadmill running kinematics

**DOI:** 10.1186/s13102-023-00725-0

**Published:** 2023-09-22

**Authors:** Ozan Sever, Rıdvan Kır, Cihan Baykal, Zeki Akyildiz, Hadi Nobari

**Affiliations:** 1 Sports Science Faculty, Atatürk University, Erzurum, Turkey; 2 Physical education and sports department, Necmettin Erbakan University, Konya, Turkey; 3 Sports Science Department, Gazi University, Ankara, Turkey; 4 Department of Exercise Physiology, Faculty of Educational Sciences and Psychology, University of Mohaghegh Ardabili, Ardabil, 5619911367 Iran; 5 Faculty of Sport Sciences, University of Extremadura, Cáceres, 10003 Spain

**Keywords:** Overhead Squat Assessment, Running kinematics, Running Gait, Lower extremity, Movement Screening

## Abstract

**Purpose:**

Overhead squat assessment (OHSA) is a pre-activity dynamic movement analysis tool used to define deviations from an ideal motion pattern which known as compensation. Compensatory movements may result from abnormality in myofascial activity, length-tension relationships, neuro-motor control strategies, osteokinematics and arthrokinematics. The aim of this study is to identify the association between selected biomechanical variables of the ankle, knee, hip, pelvis, torso during OHSA and 16 km/h treadmill running tasks.

**Methods:**

Thirteen national long distance male runners (17.3 ± 0.5 age (years); 5.89 ± 1.95 experience (years), 57.9 ± 3.7 body mass (kg); 175.4 ± 5.7 height (cm)) participated in this 2controlled laboratory study. Three-dimensional kinematics were collected at 250 Hz using a 9-camera Qualisys motion analysis system (Qualisys AB, Goteborg, Sweden) while participants performed 16 km/h treadmill running and OHSA tasks.

**Results:**

Correlation coefficients demonstrated that OHSA pelvic anterior tilt angle was in a positive association with foot strike (FS), mid-stance (MS), and toe-off (TO) pelvic anterior tilt angles and MS tibial internal rotation on talus, MS ankle pronation, MS hip internal rotation. OHSA pelvic anterior tilt angle was in a negative association with TO hip extension. OHSA maximal hip adduction was positively correlated with MS and stance maximal knee adduction. FS, MS, stance maximal angular dorsiflexion values were positively correlated with OHSA dorsiflexion. Increased OHSA dorsiflexion angle was negatively associated with TO plantar flexion. OHSA pronation was positively associated with MS and stance pronation. MS hip internal rotation, MS hip adduction angles were increased, and MS ankle dorsiflexion was significantly decreased with the increase of trunk forward lean relative to tibia during OHSA.

**Conclusions:**

OHSA was associated with some important and dysfunction-related hip, knee and ankle kinematics. Running coaches, may use OHSA as an assessment tool before the corrective training plan to detect injury-related compensation patterns to reduce the risk of injury and improve running technique.

## Background

The risk of injury in running is common. The incidence of running injuries ranging from 19.4 to 92.4%, and prevalence ranging from 6.8 to 59 per 1000 h of exposure to running [1–3]. Most running injuries are lower extremity injuries, with a predominance for the knee injuries [2, 4]. Injuries are associated with the balance of external forces acting on the human body (ground reaction forces and inertial forces created by moving body segments) and internal forces created by muscles, tendons, ligaments and joint capsules [5]. Acute injuries may occur if the external forces acting on a particular anatomical structure are higher than the internal forces. In addition, although the internal forces can manage to balance external loads, external forces that exposed for a long time with a certain cycle can cause chronic injuries [6]. About 50 to 75% of all running injuries appear to be overuse injuries due to the constant repetition of the same movement [4].

One of the goals of studying movement patterns during running gait is to gather information that can help reduce rates of injury. A thorough understanding of optimal walking and running gait is essential to eliminate dysfunctions, prevent injuries, and improve running performance. Biomechanical patterns, muscular strength, anatomical alignments, tissue flexibility and joint mobility-stabilization abilities are primarily studied topics examined in terms of chronic overuse injuries of runners. Although the abnormalities in running kinematics are partially related to some of the injuries, the underlying causes of the injuries have not been fully explained. Efficient movement patterns can contribute to reduce impact forces, ensure proper coordination between muscle activity and optimal joint movements which may reduce injury possibility, increase running economy and performance [7].

To analyze motion defaults some screening tools has been developed. Overhead squat assessment (OHSA) is a pre-activity dynamic motion screening tool. It is used to define deviations from an ideal motion pattern (compensatory movement) which may result from abnormality in myofascial length-tension relationships, neuromotor control strategies, osteokinematics and arthrokinematics [8]. OHSA has been commonly used in well-established screening methods such as the Functional Movement Screen (FMS) and the National Academy of Sports Medicine screening methods [9, 10]. It was found to be a valid (1.00 V-Aiken Coefficient for 14 of 15 compensations) [11] and reliable (Kappa-W = 0.76) [12] method of examining functional movement to predict injury. The multijoint nature of transitional movement in squat gives chance to assess the mobility, stability, asymmetry of ankle, knee, hip, pelvis, torso and shoulder gridle joints and segments [8].

OHSA has some advantages; for example, it is a slower movement than many other commonly used clinical screens such as a jump-landing or cutting task, making it potentially easier for clinicians to identify compensation patterns [13]. However, it can be considered as a disadvantage that it is done bipedally and does not reflect quick weightbearing movement transitions. For example maximum hip adduction angles were greater during single-leg tasks compared with double-leg tasks and greater during the faster movements (landing compared to squat) [14]. In this respect, it thought to become an important research topic whether it reflects the running which contains unipedal, faster movement transitions. Therefore, the purpose of this study was to identify the association (kinematic orientations and similarities rather than angular magnitudes) in selected biomechanical variables of the ankle, knee, hip, pelvis, torso during OHSA and running. It was hypothesized that osteokinematics and compensations occurring in OHSA will be positively correlated to those in running.

## Methods

### Participants

Thirteen national long distance male runners (17.3 ± 0.5 age (years); 5.89 ± 1.95 experience (years), 57.9 ± 3.7 body mass (kg); 175.4 ± 5.7 height (cm)) participated in this controlled laboratory study.

Sample size was estimated according to the g power analysis. During the Pearson correlation the g power was 0.80 for the thirteen participants. We performed an a priori estimation of power and sample size through the G-Power software program (version 3.1.9.6) written by Kiel University, made in Germany. The analysis is based on the Pearson correlation analysis method: power a is 0.05, and the 1-b error probability is 0.80. Sensitivity analysis was performed according to sample size. The purpose of G power analysis is to measure the power of our sample number to represent the universe. Inclusion criteria was set as no history of hip pain, no previous hip surgery, and no complaints of lower extremity or low back pain during the preceding six months. Data collection occurred in Kinesiology Laboratory of Ataturk University, Performance Measurement Evaluation and Rehabilitation Center. The study was conducted in accordance with the Declaration of Helsinki, before initiation, the participants signed informed consent to participate. Consent forms were obtained from the parents of seven athletes under the age of eighteen. The study was approved by the Atatürk University Review Board (Approval Number: 2021-3).

### Procedures

Three-dimensional kinematics were collected at 250 Hz using a 9-camera Qualisys motion analysis system (Qualisys AB, Goteborg, Sweden) while participants performed running and OHSA tasks. Totally thirty-five 14 mm diameter reflective markers used to capture pelvic, torso and appendicular segment motions. Markers were placed bilaterally to define appendicular segments of foot (forefoot 2nd and 5th metatarsal heads, heel back - calcaneus), shank (lateral malleolus, tibial tuberosity), thigh (lateral femoral epicondyle), pelvis (anterior superior iliac spine), upper arm (humeral lateral and medial epicondyles), forearm (radial and ulnar styloid processes). Coda Pelvis model [15] used to create pelvis segment by the markers installed on midpoint between posterior superior iliac spines and anterior superior iliac spines. The vertical position obtained during the static trial was then subtracted from the same position calculated during the trials. The longitudinal axis of the thigh is defined by the hip joint center and the knee joint center. The knee joint center was calculated using the running trial and a functional method [16]. Markers located on the thigh and the shank were used to calculate knee joint axis. Once the axis of rotation was found, the knee joint center was located at a predicted distance from the femur lateral epicondyle marker [17]. The thorax was defined from the shoulder acromial edge markers to the one chest marker on manubriosternal edge and two spine markers placed on spinous process of 12th vertebra, spinous process of 2nd vertebra [18]. Prior to data collection, a standing calibration trial was collected to determine the segmental coordinate systems and the joint centers.

Each participant performed 5-minute jogging for a warmup and performed familiarization trials of overhead squat task. Motion kinematics recorded for 20 s while subjects ran at the speed of 16 km/h on treadmill (H/p Cosmos Saturn® 300/100r) and subsequently three trials of overhead squat tasks performed considering the following checkpoint corrections.

**Fig. 1:**
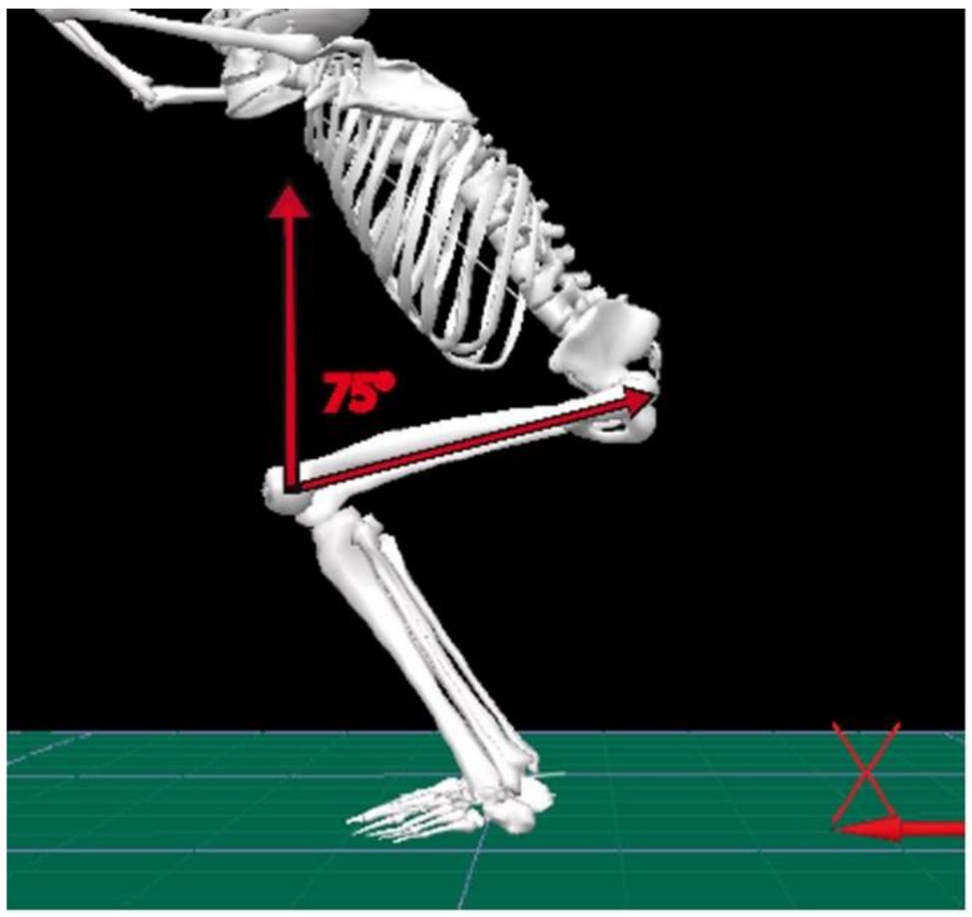
Overhead squat depth (75° squat position)

Foot position in OHSA cued as hip width (second toe directly below the anterior superior iliac spine and pointing forward) and parallel to one another. Normal lumbar curve, neutral pelvis (anterior superior iliac spines within the level of posterior superior iliac spines) was adjusted with no frontal plane asymmetry. Arms remained in line with torso with elbows locked, maintaining roughly 180° of shoulder flexion and 150° to 170° of abduction while OHSA [8, 19]. It was verbally stated that possible heel rises during OHSA restricted. No other cues gave so that compensation patterns that may occur were not eliminated. Participants were asked to choose self-selected movement speeds during the trials. 75° femur longitudinal axis - global coordinate system angle was chosen as overhead squat depth (Fig. 1) where mean heel rise was less than 3° among participants (mean 2.2° ± 1.4). The average of the three squat repetitions (each rep consists of a positive and a negative phase) for joint angles was calculated from the individual participant’s data. The individual, angular values for the participants were averaged at 75° femur-lab angle. The individual maximal angular values for the participants were averaged during the squat task between standing position to 75° squat position. Accordingly, the “Overhead Squat 75°” and “Overhead Squat Maximal” variables were created correlation analysis with the running cycle kinematics.

The movements recorded in Qualisys Track Manager software exported to Visual 3D (C-motion Inc., Kingston, Canada) to measure kinematic variables. Three-dimensional data of ankle, knee, hip joints, pelvis, and torso were calculated in running and OHSA tasks. Joint kinematics were calculated using a Cardan-Euler method in which the sequence of rotations was X (sagittal plane), Y (frontal plane), Z (vertical plane).

Time-series and trajectory patterns were normalized and averaged from foot strike (0%) to the next foot strike (100%) in 16 km/h treadmill running. The reason for choosing a relatively low running speed (16 km/h) for these athletes was to keep a load-bearing time of analyzed joints longer (longer stance phase and contact time). Angular values extracted from data series in the events of foot strike (FS), mid-stance (MS), toe off (TO) during stance phase of running. All angular velocities were calculated as the first derivatives of the angles after applying a 12 Hz lowpass filter. Maximal, mean, minimal kinematics evaluated in stance and stride phases. “Medial Knee Displacement*”* variable created to eliminate pelvis movements to exactly analyze femoral frontal plane motion. This variable represents the distance between the distal femur and the midline of the pelvis in MS phase on the frontal plane. “Overhead Squat Asymmetry*”* variable is the angle of obliquity of pelvis during OHSA. “Trunk - Tibia Angle*”* variable indicates the angle of trunk relative to tibia during OHSA.

The sign conventions of the joint angles were set as sagittal plane: flexion (+), extension (-); frontal plane: adduction (+), abduction (-), supination (+), pronation (-) and transverse plane: internal (+), external (-). Pelvic and trunk angle sign conventions set as sagittal plane: anterior tilt (+), posterior tilt (-); frontal plane: right side lower than left (+), left side lower than right (-); transversal plane: clockwise rotation (+), counterclockwise rotation (-).

### Statistical analysis

The Shapiro-Wilk test assessed the normality of data for each variable evaluated. Pearson correlation coefficient was used according to normality of distributions to evaluate the correlation of variables. Correlation magnitudes were interpreted as: trivial = < 0.1, small = 0.1–0.29, moderate = 0.3–0.49, large = 0.5–0.69, very large = 0.7–0.89, nearly perfect = 0.9–0.99, and perfect = 1. [20]

All analyses were performed with SPSS Version 21 (IBM Corporation, Armonk, NY), with an a priori level of significance of P ≤ 0.05.

## Results

Correlation coefficients demonstrated that (Table 1) pelvic anterior tilt angles were in a moderate and large positive correlation between OHSA and FS and MS respectively. A large negative correlation was found between OHSA anterior pelvic tilt and TO hip extension, MS tibial internal rotation on talus. MS ankle pronation were in a moderate association with OHSA 75°. OHSA asymmetric weight shift variables were in a large positive correlation with FS Left, MS Left and TO Right. Interestingly, OHSA transverse plane pelvic angles were in a positive correlation with running right foot pelvic rotational angles (FS and MS right with large and very large associations) (Table 1).

**Table 1:** Correlation of pelvic kinematics during OHSA and running

Pelvis							
***Sagittal Plane*** ***anterior tilt (+), posterior tilt (-)***		FS Ant. Tilt	MS Ant. Tilt	TO Ant. Tilt	TO Hip Extension	MS Hip Int. Rot.	MS Tibial Int. Rot
Overhead Squat Ant. Tilt Max.	r (% 95 CI)	0.47 (0.05–0.63)*	0.51 (0.05–0.81)*	0.39 (-0.10-0.87)	-0.56 (-0.91–0.17)*	0.58 (0.09–0.83)*	0.47 (0.13–0.69)*
	p	0.049	0.031	0.109	0.016	0.019	0.05
Overhead Squat Ant. Tilt 75°		0.54 (0.09–0.98)*	0.55 (0.03–0.90)*	0.54 (0.09–0.98)*	-0.77 (-0.90–0.40)*	0.31 (-0.22-0.72)	0.47 (0.01–0.72)*
		0.021	0.018	0.022	0.000	0.106	0.048
		FS Ankle Supination	MS Ankle Pronation				
Overhead Squat Ant. Tilt Max.	r (% 95 CI)	-0.57 (-0.24–0.83)*	0.37 (-0.12-0.86)				
	p	0.013	0.12				
Overhead Squat Ant. Tilt 75°		-0.47 (-0.11–0.85)*	0.48 (-0.05-0.77)*				
		0.046	0.044				
***Frontal Plane*** **Asymmetric Weight Shift – Pelvic Drop** ***right side lower than left (+), left side lower than right (-)***		Running Pelvic Drop at					
		FS Right	FS Left	MS Right	MS Left	TO Right	TO Left
Squat Asymmetric Weight Shift Max.	r (% 95 CI)	0.41 (-0.42-0.94)	0.78 (0.26–0.95)*	0.41 (-0.50-0.88)	0.79 (0.51–0.96)*	0.75 (0.32–0.96)*	0.23 (-0.58-0.88)
	p	0.266	0.013	0.274	0.011	0.020	0.546
Squat Asymmetric Weight Shift 75°		0.48 (-0.55-0.92)	0.82 (0.29–0.95)*	0.55 (-0.22-0.95)	0.67 (0.04–0.95)*	0.77 (0.45–0.96)*	0.30 (-0.63-0.81)
		0.194	0.007	0.125	0.048	0.016	0.433
***Transversal Plane*** **Pelvic Rotation** **clockwise rotation (+), counterclockwise rotation (-)**		Running Pelvic Rotation at					
		FS Right	FS Left	MS Right	MS Left		
Overhead Squat Pelvic Rot. Max.	r (% 95 CI)	0.78 (0.49–0.95)*	0.48 (-0.40-0.81)	0.68 (0.24–0.94)*	0.33 (-0.42-0.71)		
	p	0.013	0.205	0.044	0.381		
Overhead Squat Pelvic Rot. 75°		0.68 (0.00-0.94)*	-0.02 (-0.50-0.51)	0.28 (-0.39-0.82)	-0.20 (-0.52-0.40)		
		0.042	0.966	0.460	0.606		

FS: Foot strike, MS: mid-stance, TO: toe off phases of running. Int: Internal rotation. Ant: Anterior. Rot: Rotation. * : Significance was set at P < 0.05

According to hip joint movements (Table 2), there was one positive large association found in between running Medial Knee Displacement and Overhead Squat Hip Adduction Max variables.

**Table 2:** Correlation table of hip joint kinematics

Hip						
***Frontal Plane***		FS Add.	Stance Add. Max.	MS Add.	TO Add.	MS Medial Knee Displacement
Overhead Squat	r (% 95 CI)	0.05 (-0.47-0.58)	0.08 (-0.44-0.61)	0.18 (-0.34-0.70)	0.14 (-0.39-0.66)	0,47 (0.51–0.41)*
Add. Max.	p	0.830	0.737	0.470	0.583	0,045
Overhead Squat		0.21 (-0.31-0.72)	0.29 (-0.21-0.80)	0.23 (-0.28-0.75)	-0.09 (-0.62-0.44)	0,11 (-0.41-0.64)
Abd. Max		0.410	0.236	0.348	0.728	0,496
Overhead Squat		0.06 (-0.46-0.59)	0.14 (-0.39-0.66)	0.06 (-0.47-0.60)	-0.37 (-0.86-0.13)	0,37 (-0.29-0.75)
Add. 75°		0.799	0.586	0.812	0.135	0,130
***Transversal Plane*** **internal (+), external (-)**		FS Hip Rot.	MS Hip Rot.	TO Hip Rot.		
Overhead Squat Ext. Rot. Max.	r (% 95 CI)	0.33 (-0.16-0.83)	0.32 (-0.17-0.83)	0.24 (-0.27-0.76)		
	p	0.172	0.189	0.334		
Overhead Squat. Rot. 75°		-0.10 (-0.62-0.42)	0.28 (-0.23-0.79)	-0.13(-0.66-0.39)		
		0.691	0.257	0.593		

FS: Foot strike, MS: mid-stance, TO: toe off phases of running. Add: Adduction, Abd : Abduction, Ext: External, Int: Internal

*: Significance was set at P < 0.05

Knee joint movements demonstrated in Tables 3 and 4 (relative to femur section). OHSA knee adduction maximal angles were in a positive large association with MS and stance maximal knee adduction angles. According to relative to femur motions of tibia (knee rotation), maximal external rotation of tibia during OHSA was in a large positive correlation with MS and TO tibial external rotation angles.

**Table 3:** Correlation table of knee joint kinematics during overhead squat and running

Knee						
***Frontal Plane***		FS Add.	MS Add.	Stance Add. Max.	TO Add.	Stride Add. Max.
Overhead Squat Add. Max.	r (% 95 CI)	-0.02 (-0.51-0.51)	0.65 (0.85 − 0.25)*	0.75 (0.89 − 0.51)*	0.36 (-0.13-0.85)	0.35 (-0.28-0.98)
	p	0.925	0.003	0.000	0.143	0.148
Overhead Squat		-0.46 (-0.98–0.06)	0.34 (-0.16-0.84)	0.28 (-0.23-0.79)	-0.13 (-0.65-0.40)	-0.10 (-0.09-0.99)
Add.75°		0.052	0.167	0.260	0.617	0.687

FS: Foot strike, MS: mid-stance, TO: toe off phases of running. Add: Adduction. *: Significance was set at P < 0.05

**Table 4:** Correlation table of tibial rotation kinematics during overhead squat and running

Tibial Rotation *Transversal Plane*				
***Relative to Talus*** ***(Ankle Joint))*** ***internal (+), external (-)***		FS	MS	Stance Int. Rot. Max.
Overhead Squat Int. Rot. Max.	r (% 95 CI)	0.31 (-0.16-0.83)	0.43 (-0.03-0.91)	0.48 (0.02–0.24)*
	p	0.208	0.078	0.041
Overhead Squat Int. Rot.75°		0.21 (-0.30-0.73)	0.48 (0.11–0.79)*	0.45 (-0.02-0.93)
		0.394	0.045	0.058
***Relative to Femur*** ***(Knee Joint)*** **internal (+), external (-)**		FS	MS	TO
Overhead Squat Ext. Rot. Max.	r (% 95 CI)	0.13 (-0.65-0.40)	0.56 (-0.08-0.78)*	0.45 (-0.18-0.87)
	p	0.602	0.016	0.061
Overhead Squat. Rot. 75°		0.33 (-0.40-0.65)	0.51 (0.07–0.85)*	0.51 (-0.14-0.82)*
		0.177	0.030	0.030

FS: Foot strike, MS: Mid-stance phases of running. Int : Internal, Ext : External, Rot : Rotation

*: Significance was set at P < 0.05

First section of Table 4 demonstrates tibial rotational movements on talus and Table 5 shows ankle kinematics. Statistically significant moderate positive associations were found between OHSA Int. Rot.75° - MS and OHSA Int. Rot. Max. – Stance Int. Rot. Max. variables.

**Table 5:** Correlation table of ankle joint kinematics during overhead squat and running

Ankle						
***Sagittal Plane***		FS DorsiFlx.		MS DorsiFlx.	Stance DorsiFlx. Max.	TO PlantarFlx.
Overhead Squat DorsiFlx. Max.	r (% 95 CI) p	0.48 (0.09–0.6)*	0.71 (0.22–0.89)*		0.77 (0.25–0.89)*	-0.54 (-0.80-0.17)*
		0.044	0.001		0.000	0.021
Overhead Squat DorsiFlx. 75°		0.50 (0.16–0.75)*	0.74 (0.30–0.90)*		0.79 (0.30–0.92)*	-0.57 (-0.81-0.13)*
		0.035	0.000		0.000	0.013
***Frontal Plane***		FS Supin.	MS Pron.		Stance Pron. Max.	TO Supin.
Overhead Squat Pron. Max.	r (% 95 CI)	-0.21 (-0.31-0.73)	0.51 (0.05–0.96)*		0.48 (0.15–0.70)*	0.03 (0.56 − 0.50)
	P	0.408	0.030		0.042	0.919
Overhead Squat Supin. Max.		0.48 (-0.15-0.94)*	-0.46 (-0.93-0.04)		-0.50 (-0.69–0.32)*	0.23 (-0.28-0.75)
		0.044	0.052		0.034	0.35
Overhead Squat. 75° Pron.		-0.28 (-0.23-0.79)	0.52 (0.06–0.97)*		0.48 (0.23–0.73)*	-0.12 (-0.41-0.64)
		0.266	0.028		0.045	0.646

FS: foot strike, MS: mid-stance, TO: toe off phases of running. Flx : Flexion, Pron : Pronation, Supin : Supination

*: Significance was set at P < 0.05

FS, MS and Stance maximal dorsiflexion angles were positively correlated with OHSA dorsiflexion angles (moderate, very large, very large respectively) (Table 5). Interestingly, the increase in OHSA dorsiflexion angle negatively associated with the plantar flexion in TO (large association). OHSA pronation variables were correlated positively with MS and stance maximal (large and moderate respectively) pronation variables.

As it shown in Table 6, statistically significant positive very large, large associations were found between trunk-tibia angle and MS hip internal rotation, and MS hip adduction, respectively., .MS ankle dorsiflexion decreased with the increase of forward lean of the trunk relative to tibia.

**Table 6:** Correlation of trunk – tibia and trunk - pelvis angles relative to global coordinate system *(negative if trunk is upward position)* during squat and running kinematics

Trunk – Tibia Angle							
***Sagittal Plane***		MS Trunk Flx.	TO Anterior Pelvic Tilt	TO Hip Extension	MS Hip Int. Rot.	MS Hip Add.	MS Ankle Dorsi Flx.
OHSA Trunk-Tibia Angle 75°	r (%95 CI)	0.31 (-0.19-0.81)	-0.05 (-0.58-0.47)	-0.31 (-0.82-0.19)	0.62 (0.41–0.83)*	0.52 (0.00-0.84)*	-0.66 (-0.98–0.27)*
	p	0.409	0.915	0.209	0.06	0.028	0.006

FS: Foot strike, MS: mid-stance, TO: toe off phases of running. Flx : Flexion, Int: Internal, Add : Adduction, Rot : Rotation

*: Significance was set at P < 0.05

## Discussion

The aim of this study is to understand whether OHSA reflects running movements. For this purpose, the movements of the pelvis and trunk segments and the kinematics of the hip, knee and ankle joints were tried to be compared between running and OHSA. This association is discussed below with the evaluated segments and joints separately.

### Pelvis motions

As it demonstrated in Table 1 Pelvic Anterior Tilt Maximal and Pelvic Anterior Tilt 75° variables were in a large positive association with running pelvic anterior tilt movements at FS, MS, TO and, negatively correlated and restricted TO hip extension. Increased pelvic anterior tilt in OHSA appears to occur in running as well and limits TO hip extension. This finding may cause athletes to produce a lower force during the propulsion phase and increase the risk of lumbo-pelvic-hip complex injuries. It has been suggested that anterior tilt position of the pelvis is associated with an increase in the degree of lumbar lordosis during running [21]. This compensation called “lower crossed syndrome” is common among runners. This has been proven to be associated with common lumbo-pelvic and lower extremity injuries [22, 23]. In addition, OHSA anterior pelvic tilt and excessive lordosis is associated with lower crossed syndrome [10]. Tightness of the hip flexor musculature (iliopsoas, tensor fascia lata, rectus femoris), hip joint capsule, or surrounding anterior hip ligamentous and fascial structures in runners may reduce hip extension flexibility. It has been revealed that the hip extension angle found less in individuals with increased anterior pelvic tilt [21, 24]. These findings reinforce the concept that the pelvis moves as a functional unit [25–27] (comprises sacroiliac joint, lumbar spine, and hip joint). The shortness of the hip flexors, the lack of mobility of the hip joint, underactivity of local lumbo-pelvic stabilization subsystem and posterior oblique subsystem can initiate global lumbar muscles to be used to produce thrust force in a compensatory way to increase stride length, and this contributes to the anterior pelvic tilt in running [10, 24, 28, 29].

We can also see concurrent kinematic deviations in the lower extremity with anterior pelvic tilt during dynamic movements as it shown in Table 1. OHSA anterior pelvic tilt variable was largely and moderately associated with MS Ankle Pronation, MS Hip Internal Rotation and MS Tibial Internal Rotation on talus. Anteriorly tilted pelvis creates trunk and pelvis dyssynchronism, resulting in the distal rotational abnormality of internal rotation and adduction of the femur and subsequent external rotation of the tibia on femur and pronation of the foot [29, 30]. The association between excessive anterior pelvic tilt and increased hip internal rotation, hip adduction, knee valgus and foot pronation has been mentioned before [29–31].

The asymmetric weight shift that occurs in OHSA is considered to be lateral shift of the center of gravity. Hip adduction and flexion increase in the direction that the load shifts, and the pelvis declines in this direction in frontal plane [10, 32]. This lateral deviation of the pelvis is considered the most serious deviation, as it promotes imbalance of strength and flexibility between the sides, and may result from a limitation of range of motion, proprioception deficit, pain, quadriceps strength or motor control [33, 34]. Interestingly, OHSA asymmetric weight shift variables were in a positive very large association with pelvic drop angles of FS Left, MS Left in our study. Mean running pelvis frontal plane drop was found to be 8.71° on the non-dominant leg (left leg in all runners) and 5.79° on the dominant leg in our study group. This is thought to be the source of that association. Rotation of the pelvis on the transverse plane in running was also associated with OHSA pelvic rotation (Table 1). FS Right and MS Right pelvic rotation angles were positively correlated with “Overhead Squat Pelvic Rot. Max” and “Overhead Squat Pelvic Rot 75°” variables. These results show that there was an association between pelvic drop/rotation in running and asymmetrical weight shift in OHSA. In one study, a strong positive correlation found between frontal plane motions of the trunk, hip, and knee during the midstance phase of running and stepdown test. According to researchers, the stepdown test suggested to be a useful test to use in a clinical evaluation and rehabilitation setting for runners [35].Differences in muscular strength between the limbs may be causing the asymmetry in the specified movements of the pelvis. Increased ipsilateral trunk lean, contralateral pelvic drop, hip adduction and knee abduction were observed during one leg squat in individuals with low gluteal muscle strength [36]. In one study, individuals displaying a lateral hip shift during squat presented with less hip abduction and decreased gluteus medius activation on the limb shifted toward compared to the control group. Additionally, lateral hip shift group had greater internal rotation and less dorsiflexion on the limb shifted toward compared to the limb shift away from [32]. The revealed correlation between asymmetrical weight shifting during OHSA and stance phase pelvic drop observed in the non-dominant leg was an important finding in our study and may indicate the importance of trainers to work on the strength of the non-dominant leg hip abductors.

### Hip kinematics

Only statistically significant correlation found in hip kinematics was between Running Medial Knee Displacement and Overhead Squat Adduction Max. variables (Table 2). The variation in the motion of the pelvis on three planes during a one-leg running task may cause the femur-pelvis angle to differ significantly compared to the bipedal squat. Therefore, “the distance between the center of the pelvis and the distal lateral femur” was created as another variable (Medial Knee Displacement) to determine hip adduction in running. A positive moderate association between this variable and the Overhead Squat Add. Max. was revealed (Table 2). Similarly in one study, significant positive association of hip adduction angles were observed between single leg landing - double leg landing and single leg squat tasks [14]. Hip adduction has been linked to greater hip internal rotation during squat and step-down tasks [37].

### Knee and ankle kinematics

Dynamic knee valgus was previously defined as the three-dimensional motion of the distal femur toward and distal tibia away from the midline of the body [38]. Clinical assessment of dynamic knee valgus is usually performed through visual appearance of medial knee displacement (MKD) during the overhead squat [39]. Knee adduction/abduction were the analyzed knee motions in our study to understand knee valgus/varus in which distal tibia moves medially/laterally relative to femur in frontal plane. As it shown in Table 3, OHSA knee frontal plane kinematics were in a large association with MS and stance knee kinematics. Previous studies also demonstrated in runners [40] and females [41] that increased knee valgus during single leg squat was in a positive association with running and single leg landing knee valgus. For the variables of maximum knee abduction, maximum hip adduction angles, and maximum external knee abduction moments, positive correlations were observed between the single leg landing - double leg landing and single leg squat tasks [14]. Atkin et al. (2014) identified a moderate correlation for 2D knee abduction angles between a single-leg landing and a single-leg squat [41]. These studies reveal that frontal plane knee movements are associated with bipedal and unilateral landing, squat, and running tasks.

Relative to femur tibial rotation (knee rotation) is another knee motion in which angular value was measured between tibia and femur in transversal plane. In our study, OHSA knee rotation angle was in a large positive correlation with MS knee rotation (Table 4). In other words, increasing external rotation during OHSA was associated with MS external rotation where this angle reaches the highest value during running phases. Similarly in previous studies, it was explained that the external rotation of tibia during squat had an association with increased femoral internal rotation, hip adduction, medial knee displacement, knee abduction, ankle pronation and reduced dorsiflexion [39, 42, 43].

Transversal plane movement of the tibia relative to the talus is associated with subtalar joint supination and pronation. Accordingly, pronation movement causes internal rotation of the tibia relative to the talus, and supination causes external rotation. In this respect, tibial rotation relative to femur and tibial rotation relative to talus occur in the opposite direction of each other. As seen in Table 4, relative to talus tibial rotation was in a moderate positive association between OHSA and MS. Another association was found between Overhead Squat Int. Rot. Max. and Stance Int. Rot. Max. variables. The rotation magnitude of the tibia is affected by both the compensation in the lumbo-pelvic structure and the compensations of the lower extremities in functional movements. In closed chain movement, during the absorption phase of the stance phase of running, pronation of the foot produces internal rotation of the tibia on the foot [44]. According to that biomechanical fact, although the squat is a bipedal exercise, and knee flexion and dorsiflexion angle during squat is higher, OHSA tibial movement reflected running tibial movement.

Likewise, OHSA and running subtalar joint angles had an association. Accordingly, Stance and MS pronation angles were positively correlated with OHSA pronation variables. (moderate and large respectively, Table 5). Another important finding of our study was that the athletes with higher Overhead Squat Dorsiflexion had also **higher** FS, MS, and Stance Max dorsiflexion and **lower** TO plantar flexion angles (Table 5). In running, pronation continues until the mid-stance phase and reaches its maximum [45]. High pronation in the stance phase also means an increase in knee valgus angle and tibial internal rotation on talus [46, 47]. The synchronous actions of the knee and subtalar joint, during the contact and mid-stance phases, are interdependent motions, and the rotation of the lower leg is an obligatory action that is necessary for normal kinematics of both joints [48]. In OHSA, increased pronation is called feet flatten, and it is considered to occur as a compensation to increase the dorsiflexion angle of the ankle [19]. The reduced dorsiflexion angle and the resulting low knee flexion angle in OHSA cause some compensations movements in the foot, knee, hip joints, and pelvis and trunk segments. [19]. Decreased dorsiflexion is associated with smaller knee flexion angle, increased knee valgus, and increased ground reaction forces [49]. As knee flexion serves to lower the center of mass during squatting, limitation of this motion may result in greater frontal and transversal plane hip or knee motion as compensation [49]. Elevation of the heel from the ground, feet flatten and feet turn out are the signs of limited dorsiflexion during OHSA and can be count as a consequence of the lack of extensibility of the plantiflexers or of a foot or ankle hypomobility [34, 50]. Participants in the low-dorsiflexion subgroups exhibited greater peak hip adduction and greater peak knee external rotation compared with participants in the high-dorsiflexion subgroups during the step down test, in one study [37]. Limited dorsiflexion during weight-bearing tasks results in over pronation and tibial internal rotation to achieve additional stabilization and full body lowering [43]. Power and Clifford examined the effects of rearfoot position on squat kinematics in healthy adults with pronated feet. According to their results, the peak ankle dorsiflexion angle was significantly reduced in the group whose pronated feet were corrected to a subtalar neutral position compared to barefoot [51].

### Torso motions and trunk – tibia angle

Trunk - tibia angle variable indicates the angle of trunk relative to tibia at 75° overhead squat. The positive increase of this angle means that the trunk is tilted forward relative to the tibia. “Excessive forward lean” is accepted as a compensation pattern in OHSA. [19]. It has been suggested that this compensation occurs due to decreased hip mobility or limited dorsiflexion angle [52, 53]. More horizontal posture was adopted to compensate for changes in positioning of the lower leg and maintain the system center of mass over the base of support [53]. In our study, tibia-torso angle variable was not found to be in correlation with running torso sagittal plane movements. Sagittal plane movements occur in greater angular range in the squat. According to a study, a weak correlation for sagittal plane motion between landings and squats suggests that squats may not be sufficient for assessing sagittal plane motion during landing tasks [14]. The other finding of our study was hip, knee and ankle osteokinematics, which are suggested to related with excessive forward lean [54]. Trunk – Tibia Angle variable was in a negative correlation with MS ankle dorsiflexion and in a positive correlation with MS hip internal rotation and MS hip adduction (Table 6).

### Limitations

One important limitation of this paper is small sample size due to the relatively small number of national long distance athletes in the country where the study was conducted. Moreover, this small sample size appears to induce reduced statistical power. Despite the moderate and larger correlation coefficients, broad correlation confidence intervals can be seen in the correlation tables. Future research is required with larger sample sizes so that the confidence intervals of these correlations become smaller. This would allow a greater confidence of the strength of these relationships within similar populations of runners. Another limitation of the study; was the use of a treadmill instead of an overground running. Besides, running speed was set as 16 km/h. In fact, this running speed is quite below for the long distance race pace (over 18 km/h in a 5 K race). However, this treadmill speed was selected to see compensations by obtaining a longer stance phase during running gait cycle. Variables such as ground terrain and type and the shoes as well are known to change running kinematics. Treadmill and self-selected shoes was used in this study. It should be considered that to control aforementioned variables may change the results.

## Conclusion

The importance of hip extensors and external rotator muscles can be understood from the positive correlation between hip adduction in OHSA and MS medial knee displacement. Tibial external rotation (relative to the femur) that occurs in OHSA is positively associated with increased pronation in running in the lower extremity, according to the kinetic chain model. This finding may give us information about the trainers’ emphasis on the overactivity and shortening of the ankle pronators and knee external rotators. Besides, the dorsiflexion angles in OHSA are positively correlated with FS, MS, and stance maximal values, and negatively correlated with TO plantar flexion angle. According to this finding, those with high dorsiflexion angles TO at a lower plantar flexion angle in the propulsion phase. This relationship may affect the athletes’ foot strike patterns. Likewise, although squat and running torso sagittal plane motions are not correlated, increased forward lean in OHSA is positively correlated with hip internal rotation and adduction in running, and negatively correlated with foot dorsiflexion. In other words, low dorsiflexion may be the cause of a series of compensations in the kinetic chain from the talocrural joint to the superior joints. In this regard, low dorsiflexion mobility may need to be corrected.

Many findings revealed in the study show that OHSA is associated with osteokinematics that occur in running. In particular, the idea arises that osteokinematics, which point to many compensations that are tried to be determined by OHSA, primarily related to lumbo-pelvic region and lower extremity dysfunctions, can also be seen in running. In this regard, it may be appropriate to use OHSA as an assessment tool before the activity or before the corrective training plan for well-trained professional runners.

## Data Availability

The data presented in this study are available on website: https://osf.io/gdn5k/ with Identifier: DOI 10.17605/OSF.IO/GDN5K.
